# Computational Constraints in Cognitive Theories of Forgetting

**DOI:** 10.3389/fpsyg.2012.00400

**Published:** 2012-10-12

**Authors:** Ullrich K. H. Ecker, Stephan Lewandowsky

**Affiliations:** ^1^School of Psychology, University of Western AustraliaCrawley, WA, Australia

**Keywords:** computational modeling, consolidation, interference, decay, temporal distinctiveness

## Abstract

This article highlights some of the benefits of computational modeling for theorizing in cognition. We demonstrate how computational models have been used recently to argue that (1) forgetting in short-term memory is based on interference not decay, (2) forgetting in list-learning paradigms is more parsimoniously explained by a temporal distinctiveness account than by various forms of consolidation, and (3) intrusion asymmetries that appear when information is learned in different contexts can be explained by temporal context reinstatement rather than labilization and reconsolidation processes.

Textbooks are replete with competing explanations of why forgetting occurs. Most of these explanations are based on verbal descriptions such as “memory traces (in the phonological loop) decay over a period of a few seconds, unless revived by articulatory rehearsal” (Baddeley, 2000; p. 419). Much imaginative experimental work has gone into these verbal theories, and they have been highly influential.

Despite their success, however, verbal theories by definition remain underspecified (Lewandowsky and Farrell, 2011). They can produce testable *qualitative* hypotheses (such as “recall from verbal short-term memory must decline when rehearsal is prevented”), but because verbal theories cannot be quantitatively tested, progress is necessarily limited.

Ultimately, cognitive science needs models that make quantitative rather than qualitative predictions (cf. Lewandowsky, 1993; Farrell and Lewandowsky, 2010). If we strive for precise, specific, and falsifiable theories, if we want to understand *how exactly* theorized processes lead to an observable outcome, then we must rely on computational models.

The advantages of computational modeling are manifold and have been reviewed elsewhere (Cavagnaro et al., in press; Fum et al., 2007; Lewandowsky and Farrell, 2011). Perhaps most important, computational models “force” the theorist to be specific and explicit on how their theory actually works, avoiding the vagueness, and conceptual gaps that verbal theories allow. For example, in a verbal theory such as the phonological loop, virtually any amount of forgetting (or lack thereof) can be explained by the “right” combination of decay and rehearsal. Whenever forgetting is absent, rehearsal was able to counteract decay, and whenever forgetting is present, there was insufficient rehearsal. In other words, unless one specifies the rate of decay and the shape of its function, almost any outcome can be explained by the same model. Moreover, even when such quantitative constraints are sought, they turn out to be difficult to implement: The phonological loop, for example, can be implemented in at least 144 different computational models – depending on decisions about how decay and rehearsal operate – which produce a wide range of predictions (Lewandowsky and Farrell, 2011). All of those difficulties are avoided when a theory is instantiated in a computational model.

We underscore the value of computational modeling for theorizing in cognition with three recent examples from the area of memory and forgetting.

## Forgetting in Short-Term Serial Recall: Decay vs. Interference

The “standard” model of short-term memory (cf. Nairne, 2002) assumes that information held in short-term memory is quickly forgotten unless it is constantly rehearsed or refreshed. This is because information is thought to decay over time. Baddeley’s working memory theory is one of the theories emphasizing decay-based forgetting (Baddeley and Hitch, 1974; Baddeley, 1986, 2000); Barrouillet’s time-based resource-sharing theory (TBRS; discussed below; Barrouillet et al., 2004, 2007) is another. Decay is assumed to be a constant force, meaning that in the absence of rehearsal or refreshing^1^, a certain amount of time equates to a certain amount of trace decay.

Other models of forgetting, in contrast, have stressed that it is not time (viz. decay) *per se* that produces forgetting, but activities that – when they occur – require time to execute, where those activities interfere with retrieval of the memoranda (cf. Underwood, 1957; Anderson and Neely, 1996; Wixted, 2004). Recent examples of this kind of theory include Oberauer and Kliegl’s theory of working memory capacity limitations (Oberauer and Kliegl, 2006), Farrell and Lewandowsky’s (2002); Lewandowsky and Farrell (2008) theory of serial recall (implemented in the computational model SOB, discussed below), as well as temporal distinctiveness theory (implemented in the computational model SIMPLE, also discussed below; Brown et al., 2007).

These two rival accounts of forgetting – decay and interference – have long co-existed (cf. Wixted, 2004). While some researchers have recently concluded that a solid case has been made against decay (Berman et al., 2009; Lewandowsky et al., 2009; Jalbert et al., 2011), the notion of decay continues to be popular (Alt and Spaulding, 2011; Barrouillet et al., 2011). Enter computational modeling.

Oberauer and Lewandowsky (2008) compared computational instantiations of decay- vs. interference-based theories, applying them to data from a series of experiments that controlled rehearsal and featured distractor stimuli in addition to the memoranda (mostly complex-span tasks, which intersperse encoding of memory items and some secondary processing task). Oberauer and Lewandowsky found that the two decay-based models they tested – the “primacy model” (an implementation of the phonological loop; Page and Norris, 1998) and a “positional decay model” (cf. Burgess and Hitch, 1999; Daily et al., 2001) – invariably underestimated the effects of distractors but consistently overestimated the effects of temporal delays. That is, the data suggested that the actual amount of forgetting left unexplained by factors other than time is smaller than must be assumed by decay models.

In contrast, a model implementing interference-based forgetting (SOB; Farrell and Lewandowsky, 2002; Lewandowsky and Farrell, 2008) accounted well for the data. SOB is an associative network model that binds distributed item and positional-context representations, with no role of time in forgetting. Forgetting in SOB is instead interference-based: Because items are associated to overlapping context markers, they tend to over-write each other during encoding into the common associative network.

More recently, Oberauer and Lewandowsky (2011) computationally implemented one of the most successful verbal theories of complex-span performance, the TBRS (Barrouillet et al., 2004, 2007). This theory attributes forgetting to decay, which occurs during distractor processing during the complex-span task. Decay is counteracted by attentional refreshing, which like articulatory rehearsal restores memory traces during gaps in between distractors. One of the main predictions of TBRS is that forgetting depends on cognitive load, viz. the balance of the time for decay and refreshing.

The computational instantiation of TBRS – called TBRS* – was able to handle most benchmark findings from the complex-span paradigm, which at first glance provides both a validity check and a sufficiency proof for the verbal theory: The theory is coherent, its implementation (largely) produces the expected predictions, and it can explain a wide variety of empirical findings. However, the computational implementation also revealed some unexpected departures from the verbally derived predictions: Specifically, in contrast to the verbally derived prediction of the TBRS, the modeling demonstrated that the number of distractors in between pairs of memoranda can affect memory performance even when cognitive load is held constant. This is because when cognitive load is high – that is, when there is more time for decay (during processing of a distractor) than for refreshing (after processing of a distractor) – refreshing will no longer be able to completely reverse the effects of decay, and this will be aggravated by increasing the number of distractors.

A recent study (Oberauer et al., 2012) compared TBRS* with the most recent version of the SOB model in their application to a range of benchmark phenomena in the complex-span paradigm. Oberauer et al. examined phenomena including serial position curves, the distribution of recall errors, and the effects of cognitive load and of the number and similarity of distractors. Across a range of simulations, SOB’s fit to the data was equivalent or superior to the fit of TBRS*.

In summary, the modeling with TBRS* demonstrated that TBRS is a solid and useful theory, but it also revealed shortcomings and discrepancies between the theory’s actual behavior and verbally derived predictions. None of those would have been obtained by verbal theorizing alone.

## Forgetting in the Long-Term: Consolidation-Failure vs. Temporal Distinctiveness

In the field of neuroscience, much research on forgetting invokes the idea of consolidation. Consolidation is a post-encoding neural process that is thought to inoculate memory traces against forgetting. Forgetting is thus facilitated when consolidation is disrupted by events within a certain post-encoding window, for example a brain lesion (cf. Squire and Alvarez, 1995), certain drugs (cf. McGaugh, 2000), or some taxing mental activity requiring much cognitive resources (cf. Wixted, 2004). Generally, any period of relative inactivity following learning – that is, any period allowing consolidation to fully unfold its protective effects – will benefit memory. The hallmark of all behavioral data offered in support of consolidation is hence an improvement of memory as time between encoding and disruption increases. Consolidation has been used to explain the beneficial effects of 30-min post-encoding rest (Cowan et al., 2004), a night’s sleep (e.g., Walker et al., 2003), and also the fact that memory impairments in dementia and retrograde amnesia depend on recency, with more recent memories most affected (i.e., the Ribot gradient; e.g., Squire, 1992).

However, much like verbal views of decay-based forgetting from working memory, consolidation as a process remains underspecified (but see below). The rate of consolidation, its functional form, and in particular its time-scale remain unclear. This must be of concern because – just like with decay and refreshing or rehearsal – in principle any empirical result can be explained with the “right” combination of forgetting and consolidation.

Consolidation theorists differentiate between a short-term synaptic consolidation process and a longer-term system consolidation process, although the exact time-scale of both these processes is unclear. Estimates for the former process range from milliseconds to hours, and for the latter from minutes to decades (Dudai, 2004; Miller and Matzel, 2006). One of the obvious questions is: How can a process be finalized in one case after, say, 28 days (cf. Dudai, 2004; Figure 1), but not be finalized after many years in another (as suggested by the Ribot gradient in retrograde amnesia; e.g., Squire, 1992)? One obvious putative answer is that system consolidation itself may not be a unitary process, and a further differentiation may be needed (as suggested by, e.g., Meeter and Murre, 2004). While this differentiation may be necessary and plausible, it does open the door to a potentially infinite regress in which more and more distinct types of consolidation are needed to explain the data, depending on the particular time-scale of the experiment. The other possible response, therefore, is the more radical suggestion that a consolidation process may not be needed at all to explain the data.

As discussed by G. Brown and Lewandowsky (2010) and demonstrated by Lewandowsky et al. (2012), much of the behavioral data used to support consolidation theory – for example, the temporal gradient of retroactive interference – can be parsimoniously accounted for by a computational model of memory (SIMPLE; Brown et al., 2007) that is based on the principle of temporal distinctiveness and contains no consolidation mechanism.

SIMPLE assumes that memory items are represented in a multidimensional mental space. One of these dimensions represents time, and time-of-encoding can be used as a retrieval cue, in particular if encoding is recent. The more an item is isolated in psychological time – the greater its temporal distinctiveness – the less interference there is from neighboring items, and the more readily it is therefore retrieved. Hence, although SIMPLE predicts memory performance from temporal parameters, forgetting is assumed to be caused by interference with no causal role of time itself (i.e., decay). SIMPLE can explain much of the behavioral data taken to support consolidation by the fact that the period of mental inactivity during which consolidation purportedly takes place renders the preceding memoranda more temporally isolated, and hence more retrievable.

One important aspect of this model is its time-scale invariance. This means that absolute time is irrelevant for the model; what matters is relative time. The model will predict equivalent recall if two encoding events are spaced 1 min apart and the retention interval is 10 min, or if the events are spaced 1 h apart and the retention interval is 10 h. It follows that forgetting across various time-scales can be explained more parsimoniously by the single principle of temporal distinctiveness without reference to multiple types of consolidation (see Lewandowsky et al., 2012).

The fact that SIMPLE can explain some of the results often cited in support of consolidation does not speak against the existence of consolidation^2^ – however, it creates a quantitative benchmark against which any notion of consolidation must be evaluated. At present, consolidation is used as a ubiquitous explanans without being adequately constrained. In consequence, the consolidation notion has been over-extended to situations in which a parsimonious alternative explanation exists (cf. also Rickard et al., 2008).

Attempts to implement consolidation into computational models of forgetting are therefore particularly relevant. For example, McClelland et al. (1995) as well as Meeter and Murre (2004, 2005); Murre (1996) have used connectionist models to implement long-term system consolidation, in particular the presumed process by which memories become independent of the hippocampus over time. These models implement system consolidation as a gradual learning process, strengthening “intra-cortical” connections guided by a “hippocampal” trace reinstatement process.

Both models suggest several constraints on consolidation theorizing: (1) System consolidation must be slow and (2) interleaved with presentation of new activation patterns, in order to avoid “catastrophic interference” with existing memories (McClelland et al., 1995). This explains why system consolidation must operate on a long time-scale. Also (3) the selection of a pattern for consolidation cannot solely rely on the pattern’s strength-of-activation in “neocortex” (e.g., it could also depend on the “hippocampal” input) in order to avoid excessive and exclusive consolidation of the strongest “intra-cortical” memory traces (“runaway consolidation”; Meeter and Murre, 2005).

These models offer insights into the neuropsychological mechanisms that might govern system consolidation, and have been successfully applied to data from amnesic patients. However, to the best of our knowledge, this work has not been used to systematically constrain consolidation theorizing in non-clinical forgetting and on shorter time-scales. In particular, those models do not contain the scale invariance that imbues SIMPLE with its ability to handle the data from numerous interference experiments on different time-scales.

## Memory Updating: Reconsolidation vs. Temporal Distinctiveness

A similar case about over-extension can be made in the context of reconsolidation, a presumed manifestation of consolidation not at initial encoding but during a later episode at which an earlier event is retrieved. Neuroscientists have proposed the processes of labilization and reconsolidation to explain the fact that memories can still be updated, distorted, or even erased after they have been consolidated for considerable time. The theory goes that a memory trace (after initial consolidation) reenters a labile state when it is retrieved, and that this labilization is a prerequisite for any modification of the memory trace (e.g., updating). The labilized memory trace must then be reconsolidated in order to restabilize it in its updated form (see Hardt et al., 2010, for a review, and Osan et al., 2011, for a neural network model of reconsolidation).

One of the suggestions made by advocates of reconsolidation theory has been that reminders of the initial study context can serve to activate and hence labilize memories, making them prone to distortion (Hupbach et al., 2007, 2009). In these studies, people consecutively studied two lists of items in different contexts (e.g., in different rooms using different set-ups). Reminding people of the first context (e.g., by mentioning a particular apparatus used during study 1 in context-1) before study of the *second* list impaired memory for the *first* list – presumably because list-1 memory was labilized by the context-1 reminder, making it susceptible to change, and hence leading to list-2 intrusions into recall of list-1, but not vice versa.

The question arises whether the explanation of this curious intrusion asymmetry requires reconsolidation theory. As noted by Sederberg et al. (2011), there are a number of sophisticated computational models that can explain many fundamental properties of episodic memory, none of which make any reference to reconsolidation.

Sederberg et al. (2011) applied the Temporal Context Model (TCM; Howard and Kahana, 2002; Sederberg et al., 2008) to the data of Hupbach et al. (2007, 2009), in order to ascertain if there was a viable alternative explanation for the asymmetry, or conversely, whether the TCM model might have to be amended to include a reconsolidation mechanism.

TCM is a connectionist model with two layers: an item-layer, coding for the memory items and some contextual information (e.g., spatial information, other items present at encoding), and a temporal context layer. Temporal context is conceptualized as item-layer information that has been abstracted over time; that is, temporal context information is a recency-weighted running average of the item-layer information. In simple words, episodic encoding in TCM involves binding items to their temporal encoding context. Retrieval involves cueing with a temporal context, which then reinstates a memory item (which will then lead to further reinstatement of the item’s temporal study context, which in turn can be used as a cue for the retrieval of additional items, and so on). There is no implementation of labilization or restabilization processes in TCM.

Sederberg et al. (2011) found that the asymmetric pattern of intrusions reported by Hupbach et al. (2007) falls naturally out of TCM because context-1 is not only associated with list-1 but also list-2 (because of the reminder), whereas context-2 is only associated with list-2. In TCM terms, the list-1 reminder will reinstate the list-1 temporal context. List-2 items will then be associated with both list-1 and list-2 context features. Cueing recall with list-1 context will hence lead to reinstatement of both list-1 and list-2 items, whereas list-2 context will only trigger list-2 recall. TCM achieved an excellent quantitative fit of the data of Hupbach et al. (2007), demonstrating that the intrusion asymmetry can be parsimoniously explained by item-context binding and contextual reinstatement, without any of the assumptions of reconsolidation theory.

Support for reconsolidation has also been drawn from procedural/implicit memory experiments employing dual study events. Unlike Hupbach et al. (2007, 2009), these studies did not give subtle context reminders before the second study event, but reexposed their participants directly to the initially studied content. For example, participants in Walker et al., 2003; Experiments 5–8) performed a learned finger-tapping sequence before studying a second sequence, and participants in Schiller et al., (2010) were reexposed to a conditioned stimulus pairing before extinction of that conditioned association. In both cases did the reexposure impair memory for the initial study content. An application of a TCM will be less straightforward in these instances, so these results provide stronger *prima facie* support for reconsolidation. However, to the extent that any reexposure to a distinct studied content will reinstate its study context, alternative explanations within a temporal context framework may also be feasible, although the precise details remain to be worked out by future research.

## Conclusion

For a cognitive model to be maximally useful, it has to be computationally precise and it must quantitatively predict performance across a wide range of tasks. Modeling can thus be very beneficial for theorizing in cognition. Verbal theories of cognition can only lead to true progress in our understanding of the mind if they are ultimately specified to a degree that allows their computational implementation.

While verbal theorists may argue that computational models do not incorporate all of the strategies or metacognitive knowledge that a person can use during retrieval, this does not by itself endorse the use of verbal theorizing. Any aspect of a verbal theory should – in principle – be specifiable in a computational model, and this includes strategies and metacognitive knowledge or indeed any other construct. Without such specification, it will necessarily remain unclear how any given aspect of a verbal theory contributes to its explanatory power.

At the other extreme, researchers who create models at the neuronal level may argue that computational models of cognition will need to implement not only psychological constructs but also the exact underlying neuronal processes. We argue that this would indeed be a valuable long-term goal and creating such “multi-level models” of cognition is certainly an exciting area of research (cf. Forstmann et al., 2011; Criss et al., in press).

## Conflict of Interest Statement

The authors declare that the research was conducted in the absence of any commercial or financial relationships that could be construed as a potential conflict of interest.

## Footnotes

^1^For the purposes of the present paper one can focus on the similarities between rehearsal and refreshing, even though these are conceptually and neuropsychologically separable processes (cf. Hudjetz and Oberauer, 2007; Raye et al., 2007).

^2^In fact, there is a range of neuroscientific evidence for the impact of consolidation on memory, which goes far beyond the purview of SIMPLE (e.g., the differential effects of specific sleep phases; cf. Born et al., 2006).
